# In-Depth Lipidomic Analysis of Molecular Species of Triacylglycerides, Diacylglycerides, Glycerophospholipids, and Sphingolipids of Buttermilk by GC-MS/FID, HPLC-ELSD, and UPLC-QToF-MS

**DOI:** 10.3390/ijms18030605

**Published:** 2017-03-10

**Authors:** Pilar Castro-Gómez, Olimpio Montero, Javier Fontecha

**Affiliations:** 1 Institute of Food Science Research, Spanish National Research Council (CIAL, CSIC-UAM), Bioactivity and Food Analysis Department, Food Lipid Biomarkers and Health, Campus of Autonoma University of Madrid, 28049 Madrid, Spain; mpilar.c.g@csic.es; 2 Centre for Biotechnology Development, Spanish National Research Council (CDB, CSIC), 47151 Valladolid, Spain; olimpio.montero@dicyl.csic.es

**Keywords:** buttermilk, polar lipids, phospholipids, sphingolipids, UPLC-QToF-MS

## Abstract

Buttermilk, a byproduct of butter manufacturing, has gained considerable attention due to its high concentration of polar lipids as phospho- and sphingolipids from the milk fat globule membrane (MFGM). These polar lipids (PLs) are essential components of all cellular membranes and exert a variety of indispensable metabolic, neurological, and intracellular signaling processes. Despite its importance, there are few research studies that report a comprehensive characterization of the lipid molecular species of MFGM that could contribute to a better understanding of their putative healthful activities. In this study, procedures such as pressurized liquid extraction of polar and nonpolar lipids and their fractionation by flash chromatography have been carried out. The obtained fractions were submitted to an exhaustive characterization from a lipidomic point of view. The characterization includes new data about the identification and quantification of triacylglycerides (TAG), diacylglycerides (DAG), and phospho- and sphingolipids using different chromatographic techniques. The fatty acid profile was comparable to that of the milk fat but with a highly diverse composition of fatty acids. Molecular species have also been determined by using ultra-high performance liquid chromatography/quadruple-time-of-flight mass spectrometry (UPLC/QToF-MS). The TAG (16:0/16:0/6:0) and TAG (16:0/16:0/8:0) were the predominant saturated TAG species and TAG (14:0/18:1/16:0) and TAG (16:0/16:0/18:1) presented the highest content of monounsaturated TAG species. Furthermore; over 30 molecular species of phosphatidylcholine (PC), phosphatidylethanolamine (PE), phosphatidylserine (PS), and phosphatidylinositol (PI) could be identified within PL, with PC (16:0/18:1) being the most abundant species. Whereas C16:0 was found to be the preferred FA in TAGs, it was C18:1 in PLs. Several ganglioside species have also been characterized with d18:1 ceramide moiety and secondary acyl chains ranging from C20:0 to C26:1. This approach could broaden the applications of high-resolution mass spectrometry for a better understanding of the role of MFGM and its functionality.

## 1. Introduction

The milk fat globule is formed of a core that is mainly composed of triacylglycerides (TAG) (98%–99%), which is surrounded by the milk fat globule membrane (MFGM), whose structure is defined by the relative composition of polar lipids (PLs) and proteins [1]. Furthermore, other lipid compounds such as diacylglicerides (DAG), monoacylglycerides (MAG), free fatty acids (FFAs), and cholesterol (CHOL) are currently found in different concentrations. Nowadays, milk lipid analysis is an important area of research due to the presence of bioactive FAs, such as short- and branched-chain FA and conjugated linoleic acid, in addition to other minor components, such as the PLs (phospho- and sphingolipids) [2]. Regarding the PL of the MFGM, phosphatidylcholine (PC), phosphatidylethanolamine (PE), phosphatidylinositol (PI), phosphatidylserine (PS), and sphingomyelin (SM) are the major components and incite great interest due to their potential positive effects on human health; most research is centered on some cancers and neurological pathologies [3]. In spite of the fact that the concentration of these molecules in comparison to total milk fat is low (<1% of total fat) [4], they are of great importance because the structure acquired from mammary cellular membranes provides to MFGM an exclusive PL composition in comparison with commercial PL sources [5]. Furthermore, buttermilk (BM), a byproduct obtained from butter manufacturing, accounts for a significantly increased PL content that may reach up to 20% of total lipid concentration [4,6,7]. It is remarkable that in spite of BM being known as a good source of bioactive MFGM PLs [3,8,9], the composition of this product has not been thoroughly investigated.

Recent research studies have focused their efforts on the isolation of PL using a variety of methods, tools, and techniques (thin layer chromatography, supercritical fluid extraction, micro- and ultrafiltration, etc.), as well as the use of advanced analytical techniques in different food sources to characterize their composition, which is thought to be related to their reported bioactivity in in vitro and/or in vivo experiments [3,8]. However, the mentioned techniques have some limitations: (a) they do not allow for isolating a large amount of sample; (b) the isolate is not pure enough; (c) the use of organic solvents is likely to entail a decrease in or inhibition of the potential bioactivity of the compounds; and (d) the equipment used does not allow us to carry out a comprehensive characterization of these compounds [7,10,11,12,13,14]. Therefore, in the present study other techniques such as pressurized liquid extraction (PLE) and preparative flash chromatography (FC) have been used to obtain BM lipids and isolate different BM fractions, respectively, by using food-grade solvents in an attempt to overcome the aforementioned drawbacks. PLE procedure has been previously validated for milk fat extraction and it performs a complete extraction of all lipid classes [15]. In addition, when PLE was used to obtain the BM lipid fractions, using food-grade ethanol gave a higher yield of polar lipid extracts than those obtained using non-food grade solvents, and they effectively inhibited the cell viability of the cancer cell lines [7].

Furthermore, in order to carry out a comprehensive description, these fractions have been analyzed from a wide lipidomic perspective, especially the PL compounds, through the combined use of diverse analytical techniques, including gas chromatography-mass spectrometry/flame ionization detector (GC-MS/FID) and high-performance liquid chromatography-evaporative light scattering detector (HPLC-ELSD). Ultra-high performance liquid chromatography/quadruple-time-of-flight mass spectrometry (UPLC/QToF-MS), which has the potential to substantially improve accuracy, sensitivity, and speed, has also been used for the determination and identification of intact polar and neutral lipid molecular species.

## 2. Results and Discussion

### 2.1. Fatty Acid Methyl Ester Analysis by GC-MS of Buttermilk and Isolated Fractions

Table 1 shows the fatty acid methyl esters (FAME) composition of BM fat and its lipid fractions (F1 and F2) by GC-MS. Fractions were isolated by flash chromatography (FC) using a C18 silica cartridge as described by Castro-Gómez et al. [7]. The FAME profile was comparable to the milk fat, with a highly diverse composition of fatty acids both in terms of length, (ranging from 4 to 20 carbons) and geometric conformations (branched, *cis*, and *trans*) [16,17]. Nevertheless, the BM saturated FA (SFA) content was high (76%), mainly due to its high content in myristic (C14:0), palmitic (C16:0), and stearic (C18:0) FA. The monounsaturated FA (MUFA) (23%) content was predominantly oleic acid (C18:1c9, 17%), whereas polyunsaturated FA (PUFA) was low (~1% on total FAME) and mainly accounted for by linoleic acid (LA, C18:2 c9, c12). These results are similar to those reported in previous studies in a commercial BM samples (Sokol et al. [18]; Kristensen et al. [19]).

**Figure 1:**
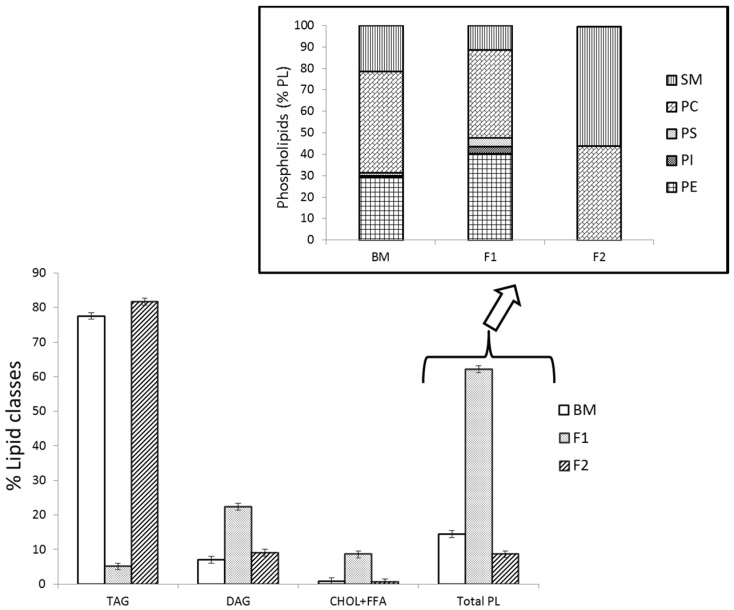
Lipid classes composition of buttermilk (BM) and isolated fractions F1 and F2 (in % of total fat) and phospholipid content (% of PL) determined by HPLC-ELSD. TAG triacylglycerols; DAG, diacylglycerols; CHOL + FFA, cholesterol + free fatty acids; MAG, monoacylglycerols; PL, polar lipids; PE, phosphatidylethanolamine; PI, phosphatidylinositol; PS, phosphatidylserine; PC, phosphatidylcholine; SM, sphingomyelin.

Differences of FA content were observed between BM and the isolated fractions F1 and F2 except for short and medium FA (from C4 to C14) (Table 1). The F1 fraction showed significantly higher PUFA content (*p* < 0.05) (0.7% vs. 2%), which has been attributed to the low content of TAG and high content of PL composition (5% and 62%, respectively) (Figure 1), in agreement with a previous study [7]. By contrast, the F2 lipid fraction presented a higher content of SFA than BM (76% vs. 84%) mainly due to the increase of the FA C16:0 and C18:0, which is also related to major neutral lipid content (82% of TAG) as can be seen in Figure 1. It is worth mentioning that the isolated F1 fraction is especially rich in phospholipids PE and PC (40% each of total PL) and the sphingolipid SM (12% of total PL), which have been shown to be beneficial compounds for human health [7,20]. These results are in agreement with the distribution reported by Smith and Lowry [21] in milk fat, who also observed an increment of SFA as well as a decrease in MUFA and PUFA in TAG when compared with the PL composition. Furthermore, this higher presence of SFA and its relation with higher TAG content and unsaturated FA to PL has also been observed in other animal fats [22,23,24,25,26]. On the other hand, Sánchez-Juanes et al. [27] and Zancada et al. [28] reported the absence of short FA (C4 to C8) in the PL fractions, while Lopez et al. [29] noted the presence of short FA in an isolated PL fraction, although at a lower concentration than was observed in the F1 fraction of this work, which could be related to the high content of DAG.

### 2.2. Triacylgliceride Analysis of Buttermilk Fat and Isolated Fractions by GC-FID

**Figure 2:**
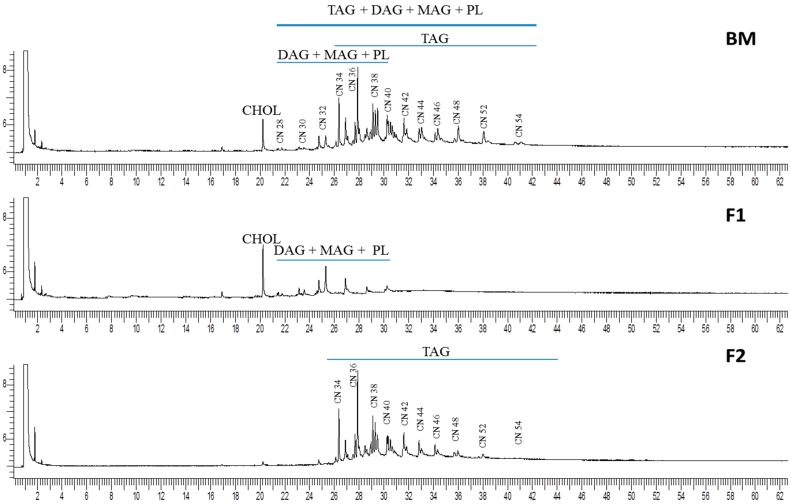
Chromatographic profile of lipid classes (DAG, diacylglycerides; CHOL, cholesterol; MAG, monoacylglycerides; PL, polar lipids) and molecular species of triacylglycerides (TAG) by carbon number (CN) of buttermilk (BM) and isolated fractions F1 and F2 determined by GC-FID.

Figure 2 shows the chromatographic profile of BM fat and its comparison with the F1 and F2 fractions as determined by GC-FID. The TAG groups were classified by their carbon number (CN) from CN28 to CN54 following previous studies [30,31]. BM and F2 samples showed a similar profile of TAG groups where CN36, CN38, and CN40 were the major TAGs, in agreement with the reported data by Fontecha et al. [30] and Castro-Gomez et al. [15] in different milk fats. However, some differences could be observed among these samples. The amount of low molecular weight TAGs (from CN28 to CN32) groups were significantly higher in BM than in F2 due to the presence of other lipid classes as DAG, MAG, and PL that co-elute with these TAGs [22] (Figure 2 F2 and Table 2). Nevertheless, the CN34 and CN36 TAG groups were higher in the F2 sample than in BM, which is related to the higher content of SFA (C16:0 and C18:0) according to the FAME analysis (Table 1). Moreover, as can be seen in Figure 2, the chromatographic profile of F1 fraction showed mainly cholesterol, DAG, MAG, and PL, which co-elute with low amount of TAG (around 5%), as shown in Figure 1. The high molecular weight TAG groups distributed from CN38 to CN54 did not show significant differences in terms of abundance between the BM and F2 fractions.

Regarding the CHOL content, the detected amount in BM sample was 0.6%, which is in agreement with the values found in other studies, ranging from 0.2% to 2%, as reported by Gille [32], Conway et al. [6], and Danthine et al. [1] in total fat and BM fat extracts. Nevertheless, the isolated F1 fraction was rich in CHOL, reaching a value of 10.2% of total fat, due to its elution in the fraction with a high amount of PL components.

### 2.3. Analysis of Buttermilk Fat and Isolated Lipid Fractions by UPLC/QToF MS

#### 2.3.1. Identification and Quantification of Triacylglyceride and Diacylglyceride Molecular Species

The isolated fraction F2 containing mainly TAG (as seen in Figure 1 and Figure 2) was submitted to UPLC/QToF-MS analysis for identification of the TAG molecular species and its FA composition. The results in Table 3 show the complexity of the TAG fraction examined, which includes a variety of molecular species from CN24 to CN54 that include double bonds (DB) from 0 to 3. The FAME content detailed earlier in Table 1 was used to ascertain the identities of the TAG, in particular when more than one TAG species with the same CN and DB was identified from the diacyl fragment ions in the high energy function (MS^E^). As can be observed, the range of CN groups identified by UPLC-QToF-MS is similar to the results obtained by GC-FID for F2 fraction (Table 2), though they only showed a TAG distribution from CN32 to CN54. This confirms the statement that the low molecular weight TAGs from CN24 to CN30 are also present in BM and F1 fraction but some of them corresponded to DAG and phospholipids isolated because of the different polarity of the solvents used for fractionation.

In this study, a total of 44 different molecular species of TAG were identified in the F2 fraction, where about 39% were fully saturated, 41% had only one unsaturation, and 20% had two or more unsaturations. These results are comparable with those reported by Calvano et al. [33], who, using MALDI-ToF-MS, observed a total of 37 different species distributed from CN 34 to CN54 (with a maximum of three unsaturations) in dairy products. In this study, TAG species with CN lower than 34 were additionally detected. Among saturated molecular species, the most abundant TAGs include those of the CN38:0 group (8.8%) and CN40 (7.6%), which corresponded with the species 16:0/16:0/6:0 and 16:0/16:0/8:0, respectively. This is in agreement with the high content of palmitic acid C16:0 in the fraction F2 (approx. 40%, Table 1), although, contrary to what might be expected, the TAG 16:0/16:0/16:0 only reached an abundance of 3.2% because most of the C16:0 are part of TAG molecular species containing other unsaturated fatty acids such as oleic acid (C18:1) as TAG 14:0/18:1/16:0 (6.2%) and TAG 16:0/16:0/18:1 (7%). It is also important to note that TAGs containing C16:0 but also C14:0 are diverted towards energetic pathways as these acyl chains are known to be the preferred acyl chains for mitochondrial β-oxidation, whereas those species containing C18:0 and C16:0 are the preferred substrate used by glycerol-3-P for de novo PL synthesis in the Kennedy Cycle [34,35]. Regarding the TAG polyunsaturated species, the most abundant were CN48:2 (16:0/16:1/16:1 and 16:1/18:1/14:0) and CN54:2 (18:0/18:1/18:1), whose abundances were 1.5%, 2.3%, and 1.5%, respectively, as has been also described by Sokol et al. [18] in a commercial BM using MS/MS.

The isolated fraction F1 containing mainly DAG and PL (as seen in Figure 1 and Figure 2) was also analyzed by the UPLC/QToF-MS. Three molecular species of DAG were identified and again the predominant compound was CN32, containing two palmitic acids 16:0/16:0 that account for 65% of the total DAG content followed by CN30 14:0/16:0 with 35% and CN32:1 14:0/18:1, which was the only unsaturated DAG species but in trace amounts. This small number of DAG species is considered normal due to the fact that they are intermediate compounds in the lipid metabolism, acting as second messengers in many cellular processes; in relatively high concentrations they can be toxic, hence they have a short life-time and are not stored in the mammary gland when milk is being produced [36,37]. These results are in agreement with those reported in milk by Calvano et al. [33], who also detected DAG with CN32 as an identifiable compound by MALDI-ToF-MS; however, the former authors also reported the presence of DAG with CN34 and CN34:1, which were not found in the present study. Similarly, Fagan et al. [38] reported 18 different species of DAG distributed from CN26 to CN36 in milk, which could be derived from lipolysis processes.

#### 2.3.2. Polar Lipid Molecular Species by UPLC-QTOF-MS

UPLC-QTOF-MS analysis, both in positive and negative mode (Table 4 and Table 5) for identification and quantification of the different PL molecular species, phospho-, and sphingolipids in the isolated F1 (which was rich in PL; see Figure 1 and Figure 2) was used.

**Figure 3:**
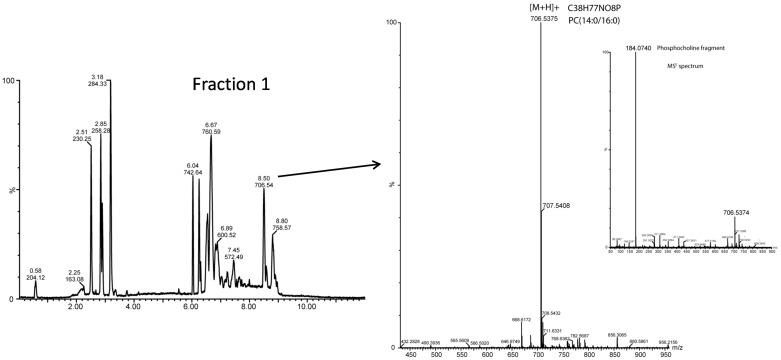
Positive ion mode spectrum of *m*/*z* 706.54 corresponding to PC (14:0/16:0) and the phosphocholine fragment obtained in the MS^E^ (high energy function) spectrum of Fraction 1 of buttermilk by UPLC-QToF-MS.

Three molecular species of lyso-PC containing the FA C16:0, C18:0, and C18:1 were identified and accounted for almost 1% of total PC species. These data are in agreement with Gallier et al. [39], who reported around 0.5% of lyso-PC from a lipid extract of BM powder using ESI-MS/MS. Furthermore, regarding the species detected, Calvano et al. [40] also reported C18:0 and C18:1 lyso-PCs, using MALDI-ToF-MS in the PL fraction of milk. Sixteen PC species were found that range in length from CN28 to CN36:3, with 28.5% saturated, 21.4% monounsaturated, and 50.1% polyunsaturated species. Very similar results were reported by Gallier et al. [39] with 23.1%, 26.3%, and 51.6% respectively, for PC from a BM analyzed with ESI/MS-MS. The predominant molecular species was C34:1 (PC 16:0/18:1), which accounted for 29.6% of the total PC content. Other PC species such as 16:0/16:0 and 18:1/18:1, along with 16:0/18:1, accounted for more than 50% of PC content. The abundance of PC (14:0/16:0) species, which reaches around 12%, is also remarkable (Figure 3). Similar results were reported by Sokol et al. [18] from a commercial BM where the predominant PC species were (16:0/18:1), (16:0/16:0), and (18:1/18:1), accounting for 25%, 11%, and 5%, respectively. Although the PC species are currently analyzed in positive mode due to the presence of the quaternary nitrogen atom with a positive charge in the choline head group, analysis in negative mode could also be carried out in the presence of a negative charge in the phosphate group and the loss of a methyl group. In negative ionization mode some molecular species of PC were not detected; however, a lyso-PC linked to C14:0 can be identified, which has not been, from our knowledge, reported before. Likewise, sphingomyelin (SM) has a positively charged quaternary nitrogen atom, which generates more abundant ions in positive than in negative mode. Nevertheless, the exact acyl chain composition cannot be determined within the present analysis and, hence, the most current species are ascribed for each *m*/*z* value. A total of six different molecular species of SM, with N-linked acyl chain distributed from C14:0 to C20:0, were identified as positive ions (Table 4). Gallier et al. [39] and Calvano et al. [40] found eight different SM species in a sample of BM powder and milk PL, respectively, but FA distribution was reported to extend from C16:0 to C24:0 and from C14:0 to C22:1. Based on the FA composition of fraction F1, the SM (d18:1/16:0) molecular specie was the most abundant, with 65% of the total SM species. This result is in agreement with Gallier et al. [39], where the SM containing C16:0 linked to the amide group was also the most abundant with a relative content of 32.4%. It is also remarkable that, despite the fact that SM linked acyl chains are known to be mainly saturated [11,27,41,42], in this study saturated species constituted less than 50% of the total content while the remaining species were exclusively monounsaturated. Finally, as occurred with the PC, the analysis in negative mode did not provide any additional information about the molecular species and confirmed that SM (d18:1/16) was the most abundant molecule with a relative content of 35%.

Other glycerophospholipids such as PE, PS, and PI, analyzed in both positive and negative ionization mode, are shown in Table 5. The [M − H]^−^ ions detected in negative mode are usually the most abundant species resulting from the breaking of the ester bond and releasing the FA as the carboxylate anion. Three molecular species of lyso-PE were identified and the acyl chain compositions of the molecular species were C16:0, C18:1, and C18:2, with a relative abundance of around 1% each. In the same way, fatty acyl residues within a total of 15 different species were identified in PE, containing seven saturated, four monounsaturated, and four polyunsaturated species distributed from C32:0 to C36:2. Among them, the species PE (16:0/18:0) in the C34:0 group was the most abundant, accounting for almost 57% of total PE. The same species of PE were identified in the negative ionization mode from [M − H]^−^ ions. These results were comparable with those achieved by Sokol et al. [18].

Three different assignments identified with both the positive and negative ionization modes of the ion signals distributed in the groups C36:0, C36:1, and C36:2 for PS are given in Table 5. Among the six possible molecular species identified, the most abundant were PS (18:1/18:1) and PS (18:0/18:2). These results suggest that the FA 16:0 was not primarily linked to PS. The absence of lyso-PS was also reported by Sokol et al. [18] and Gallier et al. [39].

Finally, although the polar head group of PI has an intrinsically negative charge, molecular species of PI were also analyzed using both positive and negative ionization modes. Six PI species with one or more unsaturations in the acyl chains within the three groups C34:1, C36:1, and C36:2 were identified. Similar results for PI were reported by Gallier et al. [39] and Calvano et al. [40] in BM and cow milk, respectively. However, Sokol et al. [18] identified more PI species, but five of the PI detected in the present study (PI 18:0/18:1; PI 18:0/18:2; PI 18:1/18:1; PI 16:0/18:1; PI 16:1/18:0) accounted for around 70% of total PI species).

In conclusion, a simple fat extraction procedure by PLE, followed by the isolation of at least two lipid fractions by flash chromatography using food-grade solvents, allowed for an exhaustive characterization of BM lipids throughout the combination of different chromatographic and lipidomic techniques. Thus, the total FA profile of the BM fat and its fractions F1 and F2 have been qualitatively and quantitatively characterized, as well as the identification of the TAG and DAG molecular species distributed from CN28 to CN54. Finally, special care has been taken to describe and identify over 30 different molecular species of phospho- and sphingolipids in the isolated polar lipid fraction from BM.

The comprehensive information obtained in this study on the lipidomic profile of BM fat is expected to be of importance to reach a better understanding of the functionality and metabolic processes in which neutral lipids and especially polar lipids like phospholipids and sphingolipids are involved in human health.

#### 2.3.3. Glycosphingolipid Molecular Species by UPLC-QTOF-MS

**Figure 4:**
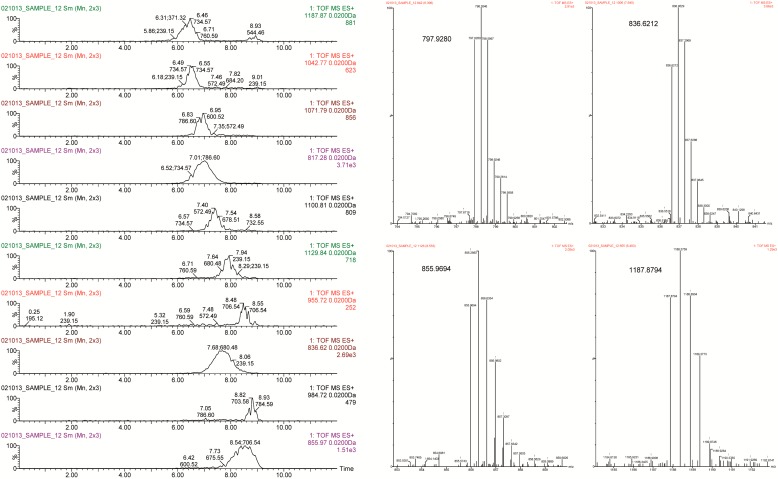
Extracted ion chromatograms (EICs) of some *m*/*z* values tentatively ascribed to gangliosides (**left** panels). Mass spectra of major m/z values with double or triple charged ions that have been ascribed to gangliosides (**right**).

Glycosphingolipid species (gangliosides) were detected with d18:1 ceramide moiety and secondary acyl chains ranging from C20:0 to C26:1 (Table 6). They all were detected as di- and/or trivalent adduct ions in the positive ionization mode (Figure 4). Even though these compounds have an anionic character, they could not be detected in negative ionization mode, which might be due to the extremely low content observed in both crude extract and fractions and enhanced ion suppression effect. In spite of these compounds having currently been analyzed in negative mode to facilitate their detection after rendering the characteristic fragment with *m*/*z* 290, which corresponds to the sialic acid (*N*-acetylneuraminic acid, NeuAc) [43], fragmentation under positive ionization has been shown to render more representative fragments of both the glycan and ceramide moieties [44]. In this study representative fragments could not be detected with the MS^E^ method and, hence, full structure identification was not feasible. Instead, the closest matching of the exact mass for the neutral molecule with annotated gangliosides in the LipidMaps database within an error of ±0.5 Da was tentatively accepted as the most probable chemical elemental composition and corresponding ganglioside species. Indeed, Lee et al. [42] also found that single MS/MS experiments did not elicit complete oligosaccharide fragmentation. Additionally, the complex structure of gangliosides is the subject of specific MS analysis, with purification by C8 SPE after extraction to remove all possible interferences [45]. Furthermore, it should be taken into account that the majority of gangliosides migrate to the aqueous phase when a partition extraction with chloroform/methanol is used. Montero et al. [46] reported a number of glycosphingolipids from ewe cheese after extraction with methanol:water (8:2, *v*/*v*); most of them were detected as multicharged ions with positive ionization rather than negative ionization. The large deviation of the experimentally determined mass from the theoretical mass may be due to the Kendrick mass defect [44].

The most abundant gangliosides from bovine milk are known to be GM3 (monosialyl) and GD3 (disialyl), in particular in the membrane fraction of the fat globule [47,48]. About seven GM and three GD are tentatively reported in the present study along with 12 neutral gangliosides (Table 6). As indicated above, since the elucidation of the exact structure was not achieved, the ascription of some gangliosides should be considered with caution.

The molecular weights of the sphingolipids noted in Table 6 are higher than those previously reported by other authors for milk or dairy products [43,44], though some coincidences were observed (compound 3 in Lee et al. [44]; compounds 2, 7, and 11 in Sørensen [43]). GD3 gangliosides were shown by Laegreid et al. [47] to predominate in bovine milk, with up to 80% of total ganglioside content; therefore, it is likely that most of the neutral gangliosides detected here could be ascribed to the GD3 after the elucidation of their structure. Lactosylceramide (d18:1/20:0) could be detected, which suggests the accumulation of this intermediate in the GM3 biosynthesis [48]. Secondary short-chain gangliosides (<C20) were not detected, which may be explained by the expected higher polarity of these compounds and complete migration towards the aqueous phase.

## 3. Materials and Methods

### 3.1. Samples and Reagents

Commercial BM powder was kindly donated by Reny-Picot (Asturias, Spain). The sample was kept frozen at −35 °C until the analysis.

All solvents were at least HPLC grade and MS grade when available. Chloroform, hexane, methanol, isooctane, isopropanol, ammonium hydroxide, arsenic formate, and acetonitrile were purchased from LABSCAN (Dublin, Ireland). Methanol (F.C.C.) ADITIO for industrial food use, sea sand, potassium hydroxide, and sodium carbonate were from PANREAC (Barcelona, Spain). Formic acid (98%) and triethylamine (99.5%), the TAG standards trinanoin and tridecanoin, the FFA standards pelargonic (C9), tridecanoic (C13), myristic (C14), palmitic (C16), estearic (C18), arachidonic (AA, 20:4), eicosapentaenoic (EPA, 20:5), and docosahexaenoic (DHA, 22:6) acids, the sterols 5α-cholestane, cholesterol (CHOL), cholesterol ester (CE), desmosterol, campesterol, β-sitosterol and lanosterol, monostearin, diolein and tripalmitin, as well as PI, PS, PE, SM, PC, and *N*-oleoylethanolamine standards were from Sigma-Aldrich (St. Louis, MO, USA). Reference samples with known composition as butter fat BCR-164 and BCR-519 (EU Com 121 missions; Brussels, Belgium) were from Fedelco Inc. (Madrid, Spain).

### 3.2. Total Fat Extraction by Pressurized Liquid Extraction System (PLE) and Lipid Fractionation by Flash Chromatography (FC)

Total fat extraction was achieved using a PLE system and the lipid extracts obtained were fractionated by a FC method, using food grade solvents (A.C.S. grade), as reported by Castro-Gomez et al. [7].

Briefly: PLE was accomplished with an ASE-200 (Dionex Corp., Sunnyvale, CA) using 2 g of BM powder sample, mixed with 2 g of sea sand. The extraction was done with food-grade ethanol during four static cycles of 5 min each. Pressure of 10.3 MPa and 60 °C temperature were used. The ethanolic extracts were filtered and gently evaporated in a vacuum rotary evaporator (Strike 202 model; Steroglass S.R.L., Perugia, Italy) and the lipid extract was afterwards weighed and stored in amber vials under a stream of nitrogen at −35 °C until analysis. Each extraction was performed in triplicate.

The lipid fractionation was carried out with a preparative Reveleris^®^ Flash System Chromatography (Grace, Deerfield, IL, USA) equipped with an ELSD. The operating conditions were set according to the manufacturer’s instructions. Briefly, an aliquot of 4 mL of BM fat (50 mg/mL) was charged in a 4 g C18 silica cartridge (Grace Reveleris, Deerfield, IL, USA). The elution solvent was methanol, followed by ethanol for 7 min at 7 mL/min each, and finally acetone for 2 min at 10 mL/min for the cleaning and regeneration of the cartridge. The lipid fractions collected were evaporated under nitrogen stream, weighed, and kept at −35 °C until further analysis. Assays were carried out in triplicate.

### 3.3. Lipid Classes Composition by HPLC-ELSD

The lipid classes analysis of BM fat and its isolated fractions was carried out as reported by Castro-Gomez et al. [7]. Briefly, a 50-µL sample was injected at 5 mg/mL in an HPLC equipment (Agilent Technologies, model 1200, Palo Alto, CA, USA) coupled to an ELSD (SEDERE. SEDEX 85 model, Alfortville, France) using pre-filtered compressed air as the nebulizing gas at pressure of 350 KPa, temperature of 60 °C, and the gain set to 3. Two Zorvax Rx-SIL columns (Agilent Technologies) of 250 mm × 4.5 mm and 5 µm particle size were used in series with a precolumn of the same packing, equilibrated at 40 °C. The solvent gradient was as detailed in Castro-Gomez et al. [15]. Assays were carried out in triplicate.

### 3.4. FA Determination and Quantification by GC-MS

The BM fat and isolated fraction samples were derivatized following the method described in Castro-Gómez et al. [16]. FAMEs were analyzed using a CP-Sil 88 fused-silica capillary column (100 m × 0.25 mm ID × 0.2 µm, Chrompack, Middelburg, The Netherlands) in an Agilent chromatograph (model 6890N) fitted with an MS detector (Agilent 5973N) that was operated in the scan mode from 50 to 550 Da. Chromatographic conditions were as in Rodríguez-Alcalá and Fontecha [17]. Briefly, the column was held at 100 °C for 1 min after injection and the oven temperature was programmed to 7 °C/min up to 170 °C, held there for 55 min, then at 10 °C/min up to 230 °C and held there for 33 min. The injector temperature was set at 250 °C. Helium was used as the carrier gas with a column inlet pressure of 30 psi. MS detector conditions were transfer line temperature 250 °C, source temperature 230 °C, and quad temperature 150 °C; electron impact ionization 70 eV. For peak identification, mass spectra obtained in our analysis were compared with those in the National Institute of Standards and Technology (NIST) library (Gaithersburg, MD, USA). The injection volume was 1 µL and split mode 1:25 was used. For qualitative and quantitative analysis, response factors were calculated using anhydrous milk fat (reference material BCR-164). Tritridecanoine (C13:0-TAG) was also used as an internal standard (200 µL; 1.3 mg/mL). Assays were carried out in triplicate.

### 3.5. Triacylglycerides and Cholesterol Determination

The samples were injected (0.5 µL at 30 mg/mL) in a CLARUS 400 gas chromatograph (Perkin Elmer, Beaconsfield, UK) fitted with a RTX-65 TAG fused silica capillary column Crossbond^®^ with 65% diphenyl:35% dimethyl polysiloxane as stationary phase (30 m × 0.25 mm ID × 0.1 µm) (Restek Corpotration, Bellefonte, PA, USA). Analysis of TAG groups by different carbon number (CN) and the CHOL contents were carried out as in Fontecha et al. 2006 [30], with the following temperature program: 120 °C, held for 30 s; 10 °C/min to 220 °C, held for 30 s; 6 °C/min to 350 °C, held for 30 min. The injector and FID temperatures were 355 °C and 370 °C, respectively. Helium was used as the carrier gas at 25 psi. For TAG identification and quantification, a reference butter fat BCR-519 was used. Samples were analyzed in triplicate.

### 3.6. Phospholipid Molecular Species Determination by UPLC/QToF-MS

Analysis of PL molecular species of the BM fat (obtained in the isolated F1 fraction) was carried out by ultra-high performance liquid chromatography (UPLC) using an ACQUITY UPLC^®^ (Waters, Manchester, UK), which was equipped with a Sample Manager model and a Binary Solvent Manager model, whose outlet was connected to an Acquity HSS T3 1.8 µm, 2.1 × 100 mm column with a pre-column of the same packing material VanGuard 1.8 µm, 2.1 × 10 mm (Waters, Barcelona, Spain). Separation was carried out with two different solvent gradients: initial, 100% A; 1.0 min, 100% A; 2.5 min 20% A; 4.0 min 20% A; 5.5 min 0% A; 8.0 min 0% A; 10.0 min 100% A; and 12.0 min 100% A. Solvent A was: MeOH:H2O (1:1) with 0.5% formic acid and 5 mM ammonium formate pH 7.5, and solvent B was: MeOH:Acetonitrile (6:4) with 0.5% formic acid and 5 mM ammonium formate. For quantification, external standards of PE, PI, PS, PC, and SM were used to draw a correlation curve of chromatographic peak area to standard concentration (µg/mL). Mass spectrometry detection of PL was carried out with a quadruple-time-of-flight mass spectrometer (QToF MS) SYNAPT HDMS G2 with electrospray ionization (ESI) source (WATERS, Manchester, UK). The chromatographic column outlet was directly connected to the ionization source. Data were acquired and analyzed with the software MassLynx^®^ (Milford, MA, USA). The sample was dissolved in MeOH:H_2_O (9:1), at 218 µg/mL and 7.5 µL was injected. The polar lipid species were detected in positive and negative modes as the [M + H]^+^ and [M − H]^−^ or [M + HCOOH − H]^−^ ions, respectively. The conditions of MS analysis were 400 to 1000 Da scanning range, capillary voltage of 0.7 V, temperature 90 °C, desolvation temperature 300 °C, gas flow 30 Lh^−1^, and desolvation gas flow 800 Lh^−1^. A MS^E^ method was operated for sample analysis, which includes a low energy function (full-scan equivalent) and a high energy function that renders fragments of the base peak *m*/*z* through a collision-induced dissociation (CID) mechanism continuously; using this high energy function, the fragment at *m*/*z* 184.074 could be monitored for PC.

### 3.7. Triacylglyceride and Diacylglyceride Molecular Species Determination by UPLC/QToF-MS

The isolated F2 and F1 fraction samples, containing mainly TAG and DAG respectively, were dissolved in a mixture of ethanol:acetone:2-propanol (1:1:1 *v*/*v*/*v*), and a volume of 7.5 µL (0.38 mg/mL) was injected using the same equipment as described above. Separation was carried out with the solvents A: acetonitrile:2-propanol:methanol (3:4:3 *v*/*v*/*v*), and B: acetonitrile:2-propanol (3:7 *v*/*v*), both with 0.1% NH_4_OH. The following elution gradient was used: initial 100% A; 3 min 100% A; 6 min 98% A; 8 min 98% A; 9.5 min 95% A; 11 min 95% A; 16 min 100% A; and 18 min 100% A. The flow rate was 0.4 mL/min. Quantification of TAG and DAG was done by drawing a correlation curve of the chromatographic peak area versus TAG (16:0/16:0/16:0) concentration (µg/mL). TAG and DAG species were detected with the ToF detector in positive mode as the [M + NH_4_]^+^ and [M + ACN + NH_4_]^+^ ions, where ACN is acetonitrile. For quantification, the [M + ACN + NH_4_]^+^ ion peak area was used. Mass spectrometer conditions were: scan from 400 to 1000 Da, capillary voltage 0.8 V, source temperature 90 °C, sampling cone 15 V, desolvation temperature 280 °C, gas flow 40 L/h, and desolvation gas flow 700 L/h. A MS^E^ method was used with low (full-scan like) and high (MS/MS-like) energy functions. Acyl groups esterifying the glycerol backbone could be identified by fragments detected in the high energy function. Independent samples were measured in triplicate.

### 3.8. Statistical Analysis

The detection of significant differences in FAMEs among whole BM and F1 and F2 fractions was carried out with a non-parametric Mann-Whitney post-hoc test. A student *t*-test between BM and F2 (rich in TAG) was used for TAG (CN groups) and cholesterol content. These statistical analysis were conducted with the aid of the SPSS package (SPSS 17.0 for Windows, SPSS Inc., Chicago, IL, USA).

## Figures and Tables

**Table 1:** Fatty acid composition (mean values and standard deviations) of buttermilk (BM) and isolated lipid fractions F1 and F2 (in %), as determined by GC-MS.

Fatty Acid	BM	F1	F2
C4:0	4.33 ^a^ ± 0.19	4.44 ^a^ ± 0.22	4.65 ^a^ ± 0.07
C6:0	2.39 ^a^ ± 0.15	2.25 ^a^ ± 0.12	2.55 ^a^ ± 0.02
C8:0	1.23 ^a^ ± 0.07	1.48 ^a^ ± 0.06	1.21 ^a^ ± 0.01
C10:0	2.70 ^a^ ± 0.10	2.73 ^a^ ± 0.01	2.63 ^a^ ± 0.06
C10:1	0.22 ^a^ ± 0.02	0.28 ^a^ ± 0.01	0.21 ^a^ ± 0.01
C12:0	3.11 ^a^ ± 0.12	3.36 ^a^ ± 0.01	3.08 ^a^ ± 0.04
C14:0	11.69 ^a^ ± 0.31	12.55 ^a^ ± 0.08	12.14 ^a^ ± 0.04
C15:0 anteiso	0.22 ^a^ ± 0.01	0.20 ^a^ ± 0.01	0.22 ^a^ ± 0.01
C15:0 iso	0.42 ^a^ ± 0.01	0.35 ^b^ ± 0.01	0.43 ^a^ ± 0.01
C14:1	0.54 ^a^ ± 0.02	0.27 ^b^ ± 0.01	0.36 ^c^ ± 0.01
C15:0	1.19 ^a^ ± 0.02	1.32 ^b^ ± 0.01	1.20 ^a^ ± 0.01
C16:0 iso	0.28 ^a^ ± 0.01	0.25 ^b^ ± 0.01	0.30 ^a^ ± 0.01
C16:0	35.39 ^a^ ± 0.05	34.47 ^a^ ± 0.34	39.96 ^b^ ± 0.09
C17:0 anteiso	0.38 ^a^ ± 0.03	0.28 ^b^ ± 0.02	0.41 ^a^ ± 0.01
C16:1	1.42 ^a^ ± 0.03	1.19 ^b^ ± 0.01	1.07 ^b^ ± 0.01
C17:0	0.63 ^a,b^ ± 0.03	0.51 ^a^ ± 0.01	0.73 ^b^ ± 0.03
C18:0	11.23 ^a,b^ ± 0.36	9.89 ^a^ ± 0.07	13.01 ^b^ ± 0.08
C18:1 t6–8	0.18 ^a^ ± 0.01	0.10 ^b^ ± 0.01	0.18 ^a^ ± 0.01
C18:1 t9	0.22 ^a^ ± 0.01	0.20 ^a^ ± 0.01	0.18 ^a^ ± 0.01
C18:1 t10	0.29 ^a^ ± 0.04	0.23 ^b^ ± 0.01	0.31 ^a^ ± 0.01
C18:1 t11	1.02 ^a^ ± 0.02	0.82 ^b^ ± 0.01	1.10 ^c^ ± 0.01
C18:1 t12	0.30 ^a^ ± 0.01	0.19 ^b^ ± 0.01	0.31 ^a^ ± 0.01
C18:1 c9	17.34 ^a^ ± 0.29	18.51 ^a^ ± 0.06	10.73 ^b^ ± 0.01
C18:1 t15	0.31 ^a^ ± 0.03	0.31 ^a^ ± 0.04	0.34 ^a^ ± 0.03
C18:1 c11	0.59 ^a^ ± 0.04	0.59 ^a^ ± 0.01	0.34 ^b^ ± 0.03
C18:1 c12	0.25 ^a^ ± 0.01	0.20 ^b^ ± 0.01	0.15 ^c^ ± 0.01
C18:1 t14 + 16	0.21 ^a^ ± 0.02	0.11 ^b^ ± 0.01	0.23 ^a^ ± 0.01
Total *trans* C18:1	2.54 ^a^ ± 0.13	1.84 ^b^ ± 0.06	2.42 ^a^ ± 0.07
C18:2 c9,c12	0.60 ^a^ ± 0.02	1.75 ^b^ ± 0.03	0.41 ^c^ ± 0.02
C18:2 c9,t11	0.03 ^a^ ± 0.01	0.09 ^b^ ± 0.01	0.15 ^c^ ± 0.01
C20:0	0.10 ^a^ ± 0.02	0.07 ^a^ ± 0.01	0.11 ^a^ ± 0.02
∑ SFA	75.96 ^a^ ± 0.55 ^a^	74.78 ^a^ ± 0.04	83.52 ^b^ ± 0.10
∑ MUFA	23.31 ^a^ ± 0.43 ^a^	23.21 ^a^ ± 0.06	15.68 ^b^ ± 0.11
∑ PUFA	0.74 ^a^ ± 0.11^a^	2.01 ^b^ ± 0.02	0.79 ^a^ ± 0.02
∑ *cis* C18:1	18.23 ^a^ ± 0.36 ^a^	19.35 ^a^ ± 0.07	11.26 ^b^ ± 0.06
∑ *trans* C18:1	2.54 ^a^ ± 0.13 ^a^	1.84 ^b^ ± 0.06	2.42 ^a^ ± 0.07

c, *cis* double bond; t, *trans* double bond; SFA, saturated FA; MUFA, monounsaturated FA; PUFA, polyunsaturated FA. Different superscript letters a, b or c in the same row mean significant differences between buttermilk and fractions F1 and F2 (*p* < 0.05).

**Table 2:** Mean values of triacylglycerol (TAG) content according to carbon number (CN) of buttermilk (BM) and isolated lipid fraction F2 determined by GC-FID.

TAG Group (%)	BM	F2	*p*
TAG CN28	1.03 ± 0.04	<0.1	0.001
TAG CN30	1.65 ± 0.10	<0.1	0.001
TAG CN32	4.29 ± 0.21	1.40 ± 0.24	0.000
TAG CN34	8.46 ± 3.20	10.90 ± 0.81	0.046
TAG CN36	10.54 ± 0.17	19.04 ± 0.98	0.004
TAG CN38	18.30 ± 0.11	16.80 ± 0.45	0.051
TAG CN40	12.53 ± 0.27	12.62 ± 0.62	0.830
TAG CN42	8.17 ± 0.41	9.17 ± 0.37	0.957
TAG CN44	7.61 ± 0.66	7.93 ± 0.30	0.494
TAG CN46	6.48 ± 0.43	6.49 ± 0.63	0.984
TAG CN48	7.69 ± 1.10	6.09 ± 0.43	0.093
TAG CN50	7.59 ± 0.67	5.99 ± 0.70	0.871
TAG CN52	5.65 ± 0.86	3.58 ± 1.08	0.742
TAG CN54	<0.1	<0.1	-

The *p*-value is given for each TAG group among BM and F2 fraction (*p* < 0.05).

**Table 3:** Triacylglicerol (TAG) molecular species content in the isolated lipid fraction F2 obtained from buttermilk ordered by carbon number (CN) and double bonds (DB) as determined by UPLC/QToF-MS.

Time (min)	Exact Mass	Content (%)	CN/DB	Molecular Species
0.93	470.3616	0.18	24:0	TAG(8:0/8:0/8:0)
0.97	578.4507	0.15	32:2	TAG(14:1/14:1/4:0)
0.98	496.3795	0.08	26:1	TAG(14:1/8:0/4:0)
1.03	498.3913	0.64	26:0	TAG(14:0/8:0/4:0)
1.07	524.4069	0.29	28:1	TAG(16:1/8:0/4:0)
1.13	526.4207	1.02	28:0	TAG(16:0/8:0/4:0)
1.45	608.4994	1.49	34:1	TAG(16:1/14:0/4:0)
1.68	636.5303	2.41	36:1	TAG(14:0/14:1/8:0)
1.72	610.5145	5.58	34:0	TAG(16:0/12:0/6:0)
1.73	662.5461	0.84	38:2	TAG(16:1/16:1/6:0)
1.96	690.5734	1.06	40:2	TAG(14:1/14:1/12:0)
2.03	638.5447	7.60	36:0	TAG(12:0/12:0/12:0)
2.40	666.5769	8.81	38:0	TAG(16:0/16:0/6:0)
2.67	772.6514	0.21	48:3	TAG(16:1/16:1/16:1)
2.67	720.6229	3.36	42:1	TAG(18:1/16:0/8:0)
2.70	694.6105	7.59	40:0	TAG(16:0/16:0/8:0)
2.95	734.6395	0.46	43:1	TAG(14:0/14:1/15:0)
3.15	774.6678	1.00	46:2	TAG(16:1/16:0/14:1)
3.18	748.6538	4.51	44:1	TAG(16:0/14:0/14:1)
3.25	722.6372	0.71	42:0	TAG(16:0/14:0/12:0)
3.53	788.6851	0.01	47:2	TAG(14:1/15:0/18:1)
3.84	802.6989	1.50	48:2	TAG(16:0/16:1/16:1)
4.71	830.7292	2.97	50:2	TAG(16:1/18:1/16:0)
3.87	776.6866	4.39	46:1	TAG(18:1/14:0/14:0)
4.30	790.7012	1.67	47:1	TAG(18:1/14:0/15:0)
4.37	764.6894	1.55	45:0	TAG(16:0/15:0/14:0)
4.71	856.7448	1.37	52:3	TAG(16:0/18:1/18:2)
4.77	804.7145	6.17	48:1	TAG(14:0/18:1/16:0)
4.87	778.6984	3.13	46:0	TAG(14:0/14:0/18:0)
5.12	818.7254	1.79	49:1	TAG(15:0/16:0/18:1)
5.19	792.7154	0.75	47:0	TAG(16:0/14:0/17:0)
5.33	818.7254	0.69	49:1	TAG(16:0/18:1/15:0)
5.43	792.7154	0.80	47:0	TAG(16:0/16:0/15:0)
5.94	832.7463	7.04	50:1	TAG(16:0/16:0/18:1)
6.05	806.7285	3.19	48:0	TAG(16:0/16:0/16:0)
6.19	828.7155	0.28	50:3	TAG(18:2/18:1/14:0)
6.22	884.7746	1.13	54:3	TAG(18:1/18:1/18:1)
6.37	872.7741	0.72	53:2	TAG(18:1/18:1/17:0)
6.40	846.7602	1.41	51:1	TAG(16:0/17:0/18:1)
6.46	820.7466	1.13	49:0	TAG(16:0/16:0/17:0)
6.81	820.7466	0.89	49:0	TAG(16:0/15:0/18:0)
7.39	834.7604	0.19	50:0	TAG(16:0/16:0/18:0)
7.50	886.7917	2.60	54:2	TAG(18:0/18:1/18:1)
7.64	860.7755	5.50	52:1	TAG(16:0/18:0/18:1)

Species detected as [M + NH_4_]^+^ and [M + NH_4_ + H_3_CCN]^+^.

**Table 4:** Phosphatidylcholine (PC) and sphingomyelin (SM) content in the F1 fraction of buttermilk as determined by UPLC/QToF-MS. The acyl chain compositions of the molecular species are shown.

Positive Mode					Negative Mode				
Time (min)	Exact Mass	Content (%)	CN/DB	Molecular Species	Time (min)	Exact Mass	Content (%)	CN/DB	Molecular Species
3.75	496.3418	0.31	16:0	Lyso-PC(16:0)	3.35	512.30	2.10	14:0	Lyso-PC(14:0)
3.93	522.355	0.24	18:1	Lyso-PC(18:1)	4.08	540.33	26.29	16:0	Lyso-PC(16:0)
4.95	524.368	0.20	18:0	Lyso-PC(18:0)	4.28	566.35	6.72	18:1	Lyso-PC(18:1)
6.53	734.5698	13.09	32:0	PC(16:0/16:0)	6.71	802.56	9.03	34:2	PC(16:0/18:2)
6.55/8.03	756.5527	1.21	34:3	PC(16:1/18:2)	6.8	828.58	1.99	36:3	PC(18:1/18:2)
6.66	760.5854	29.61	34:1	PC(16:0/18:1)	7.45	812.58	6.23	36:4/36:3	PC(O-18:2/18:2)
6.82	786.6024	9.38	36:2	PC(18:1/18:1)					/(P-18:1/18:2)
7.03	786.6024	4.71	36:3	PC(18:0/18:2)	8.27	778.56	14.12	32:2	PC(16:0/16:0)
7.56	678.5092	1.94	28:0	PC(14:0/14:0), (12:0/16:0)	8.39	804.57	23.29	34:1	PC(16:0/18:1)
7.75	730.5361	0.65	32:2	PC(16:1/16:1)	8.49	722.50	1.55	28:0	PC(12:0/16:0),
8.02/8.5	782.5671	1.72	36:4	PC(18:2/18:2)					(14:0/14:0)
8.46	706.5352	11.72	30:0	PC(14:0/16:0), (12:0/18:0)	8.52	830.59	7.13	36:2	PC(18:1/18:1)
8.55	732.5501	4.65	32:1	PC(16:0/16:1)	9.43	814.60	1.56	36:3/36:2	PC(O-18:1/18:2)
8.6	762.6018	2.56	34:0	PC(16:0/18:0)					/(P-18:0/18:2)
8.76	758.5701	8.82	34:2	PC(16:0/18:2)					
8.79	788.6178	4.95	36:1	PC(18:0/18:1)					
8.84	784.5816	4.24	36:3	PC(18:1/18:2)					
8.84	784.5816	4.24	36:3	PC(18:1/18:2)					
5.58	705.588	5.16	34:0	SM(d18:0/16:0)	6.37	747.56	34.67	34:1	SM(d18:1/16:0)
6.16	759.637	0.81	38:1	SM(d18:1/20:0)	7.47	749.58	29.64	34:0	SM(d18:0/16:0)
7.69	675.5415	14.90	32:1	SM(d16:1/16:0)	8.66	719.53	24.49	32:1	SM(d16:1/16:0)
8.19	677.5576	6.53	32:0	SM(d18:0/14:0)	9.32	721.55	11.19	32:0	SM(d18:0/14:0)
8.18	689.5562	7.92	33:1	SM(d18:1/15:0)					
8.58/8.77	703.5729	64.69	34:1	SM(d18:1/16:0)					

Species detected as [M + H]^+^ and [M + HCOOH–H]^−^. Lyso-PC, lyso-phosphatidylcholine; PC, phosphatidylcholine; CN, carbon number; DB, double bonds.

**Table 5:** Glycerophospholipid classes: Phosphatidylethanolamine (PE), phosphatidylinositol (PI) and phosphatidylserine (PS) content in the F1 fraction of buttermilk as determined by UPLC/QToF-MS. The acyl chain compositions of the molecular species are shown.

Positive Mode					Negative Mode				
Time (min)	Exact Mass	Content (%)	CN/DB	Molecular Species	Time (min)	Exact Mass	Content (%)	CN/DB	Molecular Species
3.99	480.3053	1.13	18:1	Lyso-PE(18:1)	3.53	476.2809	0.81	18:2	Lyso-PE(18:2)
7.07	718.5381	2.24	34:1	PE(16:0/18:1), (16:1/18:0)	4.09	478.2934	1.12	16:0	Lyso-PE(16:0)
7.22/7.45/8.21	744.5569	9.72	36:2	PE(18:1/18:1), (18:0/18:2)	4.3	478.2934	1.04	18:1	Lyso-PE(18:1)
7.99	692.5201	9.46	32:0	PE(16:0/16:0), (20:0/12:0), (18:0/14:0), (17:0/15:0)	6.61	688.4931	2.72	32:2	PE(14:0/18:2)
8.86/9.1	720.5502	56.58	34:0	PE(20:0/14:0), (18:0/16:0), (17:0/17:0)	7.19	740.5279	26.89	36:3	PE(18:1/18:2)
8.95	746.5662	8.70	36:1	PE(18:0/18:1), (20:0/16:1)	7.06	714.5073	9.39	34:2	PE(16:1/18:1), (16:0/18:2)
9.06	716.5188	12.14	34:2	PE(16:1/18:1), (18:2/16:0)	8.87	716.5193	15.89	34:1	PE(16:0/18:1), (16:1/18:0)
					9.06	742.5395	42.13	36:2	PE(18:1/18:1), (18:0/18:2)
6.7	790.5614	39.37	36:1	PS(18:1/18:0), (16:1/20:0)	7.45	786.5295	80.91	36:2	PS(18:1/18:1), (18:2/18:2)
6.99	792.5767	60.63	36:0	PS(16:0/20:0), (18:0/18:0)	9.39	788.5384	19.09	36:1	PS(18:0/18:1), (16:1/20:0)
8.26	854.5759	19.69	34:1	PI(16:0/18:1), (16:1/18:0)	7.76	863.5589	100.00	36:1	PI(18:0/18:1)
8.33/8.46	880.5894	80.31	36:2	PI(18:1/18:1), (18:0/18:2)					

Species detected as [M + NH_4_]^+^ and [M − H]^−^. Lyso-PE, lyso-phosphatidylethanolamine; PE, phosphatidylethanolamine; PI, phosphatidylinositol; PS, phosphatidylserine; CN, carbon number; DB, double bonds.

**Table 6:** Values of *m*/*z* from positive ionization that are tentatively ascribed to glycosphingolipid species according to the neutral mass value and the LipidMaps database (http://www.lipidmaps.org). The elemental composition that closely matches the experimental mass was chosen amongst those annotated in the LipidMaps database. *M*_exp_ is the exact mass calculated from the experimental *m*/*z* value, and *M*_Th_ is the theoretical exact mass corresponding to the proposed elemental composition.

No.	*m*/*z*	Ion	Exact Mass (M_exp_ − M_Th_)	Elemental Composition	Glycosphingolipid Class	Tentative Ceramide
1	1013.75	[M + 2H]^+2^	2025.11 (+0.37)	C_94_H_168_N_4_O_42_	Globo/Lacto/Neolacto	-Cer(d18:1/22:0)
2	1187.87	[M + 2H]^+2^	2373.23 (+0.49)	C_107_H_188_N_6_O_51_	Acidic	-Cer(d18:1/22:0)
3	984.72	[M + 2H]^+2^	1967.07 (+0.35)	C_91_H_162_N_4_O_41_	Acidic	-Cer(d18:1/22:0)
4	797.92	[M + 3H]^+3^	2390.25 (+0.48)	C_108_H_191_N_5_O_52_	Lacto/Neolacto	-Cer(d18:1/22:0)
5	875.31	[M + 3H]^+3^	2623.30 (−0.39)	C_116_H_202_N_6_O_59_	Acidic (4 isomers)	-Cer(d18:1/22:0)
6	855.97	[M + 3H]^+3^	2565.29 (−0.43)	C_114_H_200_N_6_O_57_	Lacto/Neolacto	-Cer(d18:1/20:0)
			2565.32 (−0.46)	C_116_H_204_N_4_O_57_	Neolacto (2 isomers)	-Cer(d18:1/26:1)
7	1158.83	[M + 2H]^+2^	2315.25 (+0.39)	C_107_H_190_N_4_O_49_	Acidic (3 isomers)	-Cer(d18:1/26:0)
	778.58	[M + 2H + NH_4_]^+3^	2315.25 (+0.43)	C_107_H_190_N_4_O_49_	Acidic (3 isomers)	-Cer(d18:1/26:0)
		[M + 2H + NH_4_]^+3^	2298.23 (+0.41)	C_106_H_187_N_5_O_48_	Lacto/Neolacto (2 isomers)	-Cer(d18:1/26:1)
8	836.62	[M + 3H]^+3^	2507.31 (+0.47)	C_114_H_202_N_4_O_55_	Neolacto	-Cer(d18:1/24:0)
9	955.72	[M + 2H]^+2^	1909.03 (+0.39)	C_88_H_156_N_4_O_40_	Acidic (3 isomers)	-Cer(d18:1/22:0)
10	926.70	[M + 2H]^+2^	1851.05 (+0.33)	C_88_H_158_N_2_O_38_	Globo	-Cer(d18:1/26:1)
			1851.02 (+0.36)	C_86_H_154_N_4_O_38_	Lacto/Neolacto	-Cer(d18:1/20:0)
11	1129.84	[M + 2H]^+2^	2257.21 (+0.45)	C_104_H_184_N_4_O_48_	Neolacto (2 isomers)/Acidic	-Cer(d18:1/26:1)/-Cer(d18:1/26:0)
	759.23	[M + 2H + NH_4_]^+3^	2257.21 (+0.45)			-Cer(d18:1/26:1)/-Cer(d18:1/26:0)
12	897.66	[M + 2H]^+2^	1793.04 (+0.26)	C_86_H_156_N_2_O_36_	Lacto/Neolacto (2 isomers)	-Cer(d18:1/24:0)
13	946.722	[M + H]^+^	945.7116 (+0.0025)	C_52_H_99_NO_13_	Neutral	-LacCer(d18:1/22:0)
14	1100.81	[M + 2H]^+2^	2199.20 (+0.40)	C_102_H_182_N_4_O_46_	Neolacto	-Cer(d18:1/24:0)
	739.89	[M + 2H + NH_4_]^+3^	2199.20 (+0.41)			-Cer(d18:1/24:0)
		[M + 3H]^+3^	2216.16 (+0.47)	C_100_H_177_N_5_O_48_	Lacto/Neolacto (2 isomers)	-Cer(d18:1/20:0)
15	817.28	[M − H_2_O + 3H]^+3^	2467.25 (−0.42)	C_110_H_194_N_4_O_56_	Neolacto (2 isomers)	-Cer(d18:1/20:0)
		[M + 2H + NH_4_]^+3^	2432.26 (−0.47)	C_110_H_193_N_5_O_53_	Neolacto (3 isomers)	-Cer(d18:1/24:1)
16	1071.79	[M + 2H]^+2^	2141.16 (+0.40)	C_99_H_176_N_4_O_45_	Acidic (9 isomers)	-Cer(d18:1/24:0)
	720.54	[M + 2H + NH_4_]^+3^	2141.16 (+0.40)			-Cer(d18:1/24:0)
		[M + 3H]^+3^	2158.12 (+0.47)	C_97_H_171_N_5_O_47_	Acidic	-Cer(d18:1/20:0)
17	1042.77	[M + 2H]^+2^	2083.12 (+0.40)	C_96_H_170_N_4_O_44_	Acidic (4 isomers)	-Cer(d18:1/24:0)
	701.20	[M + 2H + NH_4_]^+3^	2083.12 (+0.42)			

Values of *m*/*z* provided here were detected under positive ionization. From the observed m/z value the corresponding neutral mass value (M) was calculated by taking into account the posible ions [M + *n*H]^+*n*^, [M + NH_4_ + (*n* − 1)H]^+*n*^, [M + (*n* − 1)NH_4_ + (*n* − 2)H]^+*n*^ or [M + *n*NH_4_]^+*n*^ where *n* = 2 or 3. Ganglioside species annotated in the LipidMaps database (http://www.lipidmaps.org) that mached the calculated M within an error of 0.5 Da were considered as tentative ganglioside species corresponding to the observed *m*/*z*. The exact mass corresponding to the elemental composition of the selected ganglioside species is provided along with the error (between brackets) for the calculated M from the *m*/*z* value. *M*_exp_ is the exact mass calculated from the experimental *m*/*z* value, and M_Th_ is the theoretical exact mass corresponding to the proposed ganglioside elemental composition. The ceramide moiety is only annotated in this table because of the potential variability of the sugar moiety that was not determined.
